# Safety of African Swine Fever Vaccine Candidate Lv17/WB/Rie1 in Wild Boar: Overdose and Repeated Doses

**DOI:** 10.3389/fimmu.2021.761753

**Published:** 2021-11-30

**Authors:** Jose A. Barasona, Estefanía Cadenas-Fernández, Aleksandra Kosowska, Sandra Barroso-Arévalo, Belén Rivera, Rocío Sánchez, Néstor Porras, Carmina Gallardo, Jose M. Sánchez-Vizcaíno

**Affiliations:** ^1^ VISAVET Health Surveillance Center, Complutense University of Madrid, Madrid, Spain; ^2^ Department of Animal Health, Faculty of Veterinary, Complutense University of Madrid, Madrid, Spain; ^3^ European Union Reference Laboratory for ASF, Centro de Investigación en Sanidad Animal (CISA, INIA-CSIC), Madrid, Spain

**Keywords:** African swine fever, virus, vaccine, safety studies, wild boar, control disease, infectious disease

## Abstract

African swine fever (ASF) is a highly lethal infectious disease that affects domestic pigs and wild boar. Outbreaks of ASF have grown considerably in the last decade causing important economic consequences for the swine industry. Its control is hampered by the lack of an effective treatment or vaccine. In Europe, the wild boar is a key wild reservoir for ASF. The results of the oral vaccination trial of wild boar with Lv17/WB/Rie1 are hope for this problem. However, this vaccine candidate has certain safety concerns, since it is a naturally attenuated vaccine. Therefore, the current study aims to evaluate the safety of this vaccine candidate in terms of overdose (high dose) and repeated doses (revaccination) in wild boar. Low-dose orally vaccinated animals developed only a slight transient fever after vaccination and revaccination. This was also the case for most of the high-dose vaccinated wild boar, except for one of them which succumbed after revaccination. Although this fatality was related to hierarchical fights between animals, we consider that further studies are required for clarification. Considering these new results and the current epidemiological situation of ASF in wild boar, this vaccine prototype is a promising tool for the control of the disease in these wild populations, although further studies are needed.

## Introduction

African swine fever (ASF) is one of the most relevant infectious diseases to affect suids as regards both domestic pigs and Eurasian wild boar (*Sus scrofa*). The disease is caused by a large and complex DNA virus belonging to the *Asfarviridae* family, that evolved in south-eastern Africa in a sylvatic cycle between common warthogs (*Phacochoerus africanus*) and argasid ticks of the *Ornithodoros moubata* species complex (1). There are numerous ASF virus (ASFV) isolates, which have different levels of virulence. However, the genotypic classification, which has described 24 genotypes to date, provides only molecular epidemiological information (2).

The first known case of ASF appeared in Kenya in 1921, where it was described as a hemorrhagic disease in domestic pigs with a lethality rate close to 100% (3). After entering the Iberian Peninsula in the 1950s and its subsequent eradication in the 1990s, ASF remained confined to Africa (with the exception of the island of Sardinia) until 2007, when it entered Eastern Europe (4). Since then, ASF has remained in Eastern Europe and has spread to more countries on this continent, affecting both domestic pigs and wild boar (5). Furthermore, in 2018, ASFV first entered Asia through China, the largest global pig producer that is home to about half of the world’s pig population (6). In just two years, ASFV has spread rapidly across the Asian continent, when compared to the European scenario (5). In the last decade, ASF has generally shown a remarkable capacity for transboundary and transcontinental spread, with a growing number of outbreaks of the disease on five different continents and in more than 50 countries (5). This situation has further increased alarm and the need to control and stop the spread of ASFV.

The control of ASF is hampered by the lack of an effective treatment or vaccine, and the control measures are, therefore, based mainly on the application of strict sanitary measures that involve the depopulation of affected farms and preventive measures (7). Countries affected by ASF struggle to control and minimize losses, while countries that are still ASF free confront an increased risk of pathogen introduction. These measures have great sanitary and economic impacts, thus making ASF a notifiable disease for the World Organization for Animal Health (OIE).

In recent outbreaks, both domestic pigs and wild boar have developed the acute form of the disease, which is characterized by high fever and sudden death (7, 8). In Asia, the disease has mainly been reported in domestic pigs, with sporadic notifications in wild boar (6, 9). However, the situation in Europe is the opposite: the wild boar is the main host affected in most European countries, except for the Russian Federation, Ukraine, Romania and Serbia (5). The ASFV maintains a stable cycle in wild boar owing to the high density of this population in Europe and its ethology, such as its scavenging and cannibalistic behavior (9), since ASFV is transmitted mainly through contact with blood. The wild boar is, therefore, considered a highly relevant wild reservoir for ASF in Europe and is responsible for sporadic outbreaks in domestic pigs (10, 11).

This makes the control of the disease in wild boar even more complicated, and the strict sanitary measures traditionally employed to control ASF are, in most cases, not sufficient when the disease is widespread in wild boar populations (11). There is, therefore, enormous interest in the development of not only a safe and effective vaccine for domestic pigs but also an oral vaccine for wild boar.

Details of an oral vaccination trial carried out with wild boar as a measure against an ASFV isolate currently circulating in Europe were first published in 2019 (12). The results obtained were very promising in terms of effectiveness since they achieved 92% protection against the challenge. However, this vaccine candidate is based on a naturally attenuated virus, which raises certain safety concerns (13). This signifies that, although a practically complete attenuation was observed in the previous study, further studies to evaluate its safety are required.

Detailed requirements regarding animal vaccines are laid down in the European Pharmacopoeia and the OIE Manual of diagnostic tests and vaccines for terrestrial animals. Several studies on the immunogenicity, safety and efficacy of the vaccine candidates produced following Good Clinical Practice (GCP) guidelines in clinical trials and Good Laboratory Practice (GLP) conditions (14) are, therefore, necessary. One of the studies required is the assessment of innocuousness and safety in target species. About these safety studies, and keeping in mind that it must be possible to administer this vaccine candidate orally in wild populations in which it is more difficult to control the exact dosage, the objective of the current study is to evaluate the safety of this vaccine prototype in terms of overdose (high-dose) and repeated doses (revaccination) in wild boar.

## Materials and Methods

### ASFV Isolates

The natural attenuated non-haemadsorbing genotype II ASFV Lv17/WB/Rie1 isolate was used as a vaccine prototype. This virus had previously been described and tested in both domestic pigs and wild boar for immunization purposes (12, 15). The virus was grown in porcine blood monocytes (PBM) for 7 days, after which the culture medium containing extracellular virus was collected, and centrifuged at a low speed to remove cellular debris and then at a high speed to sediment the virus (16). Viral titer was defined as the amount of virus causing cytopathic effects in 50% of infected cultures (TCID_50_/mL) and was estimated by means of immunoperoxidase staining (17).

The highly virulent haemadsorbing genotype II ASFV Arm07 isolate was used as the challenge virus. The virus was propagated in PBM as described previously (16). Viral titer was defined as the amount of virus causing hemadsorption in 50% of infected cultures (HAD_50_/mL).

### Animals

Experiments were performed in biosafety level 3 facilities at the VISAVET Health Surveillance Centre at the Madrid Complutense University, Spain. The safety studies were carried out with 15 wild boar piglets aged 3-4 months, which were obtained from a commercial wild boar farm in Andalusia, Spain. These animals had not been vaccinated against any infectious diseases before the experiment, and tested negative to antibodies when employing the ELISA test as regards the following infectious diseases: Aujeszky virus, *Mycobacterium bovis*, classical swine fever virus, ASFV, swine vesicular disease virus, *Mycoplasma hyopneumoniae*, porcine reproductive and respiratory syndrome virus and porcine circovirus type 2. Upon arrival, all the animals were ear-tagged for individual identification and were randomly placed in the study groups. The animals were acclimatized for two weeks before the experiment began. During the acclimatization phase, the piglets received metaphylactic treatment with oxytetracycline dihydrate (Alamycin LA 300, Norbrook Laboratories, Northern Ireland) and ivermectin (Ivomec S, Merial GmbH) in order to eliminate parasites and to control any unapparent bacterial infections.

Animal care, handling and sampling procedures were conducted in compliance with regional, national, and European regulations and the *in vivo* experimental protocol was previously approved by the Ethics Committees of the Madrid Complutense University and the Community of Madrid (reference PROEX 159/19). The protocol included a detailed description of efforts to prevent and avoid the animals’ unnecessary suffering, including humane endpoints and guidelines regarding euthanasia. All procedures were designed and performed by specially trained personnel and veterinarians (animal experimentation categories B, C and D) following EC Directive 86/609/EEC.

### Experimental Design

**Figure 1:**
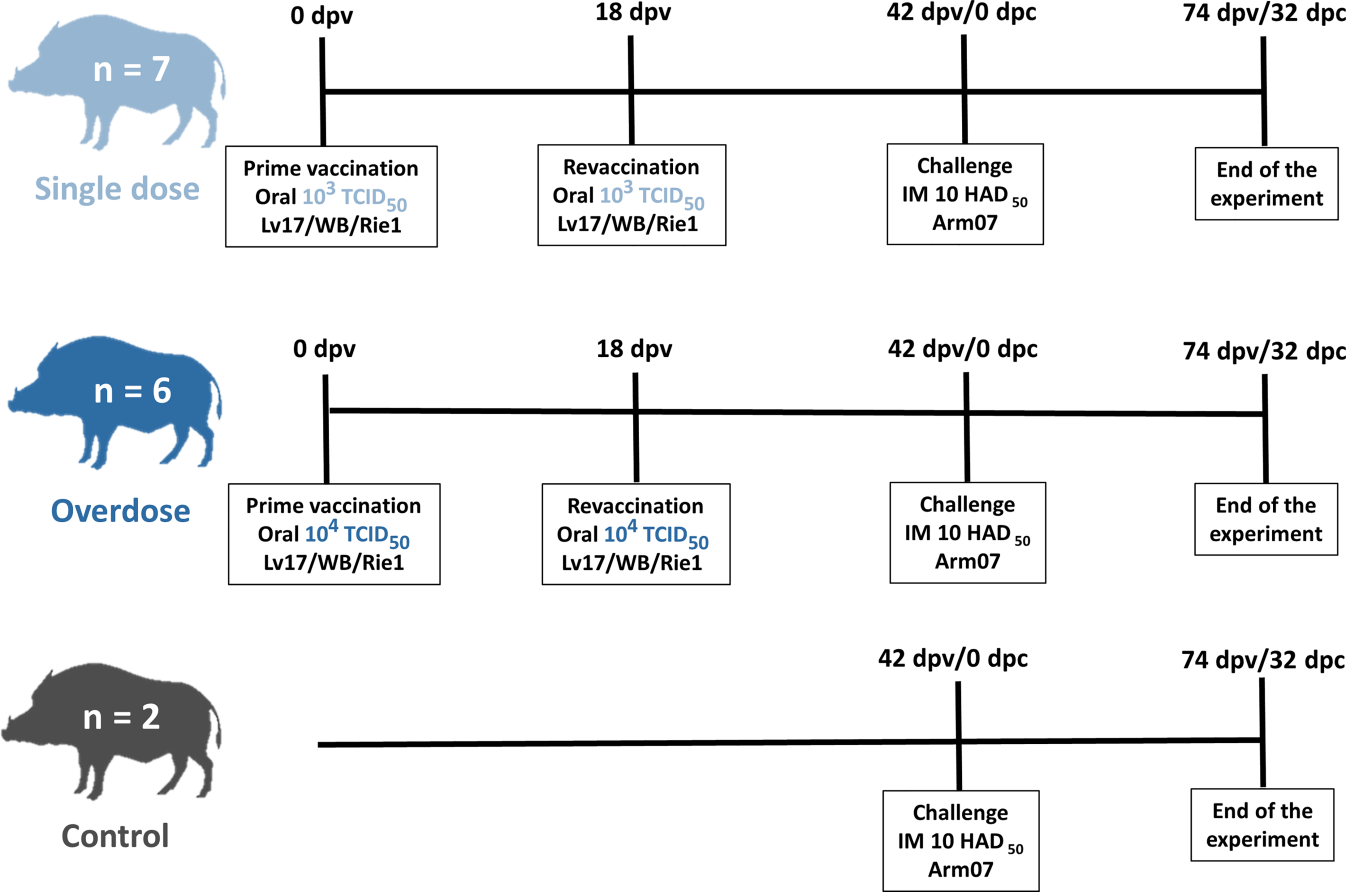
Scheme of the experimental design from the prime vaccination (0 days post-vaccination, dpv) until the end of the experiment (32 days post-challenge, dpc; and 74 dpv).

ASFV Lv17/WB/Rie1 was assessed for its innocuousness and effectiveness at different doses and during revaccination through oral administration, 10^3^ and 10^4^ TCID_50_. Oral vaccination was performed with a syringe while the animal was immobilized by the veterinary staff. Seven animals were orally vaccinated with 1 ml of 10^3^ TCID_50_ of ASFV Lv17/WB/Rie1, and these comprised the low-dose group. Another six animals were orally vaccinated with 1 ml of 10^4^ TCID_50_ of ASFV Lv17/WB/Rie1 and comprised the high-dose (tenfold) group. The two remaining animals were kept naïve until challenge, thus representing the control group. The revaccination was carried out orally at 18 days post-prime vaccination (dpv) with the corresponding administration dose, 10^3^ TCID_50_ for the low-dose group and 10^4^ TCID_50_ for the high-dose group. Forty-two days after prime vaccination (42 dpv), all the animals were exposed to the challenge through the intramuscular administration of 1 ml of 10 HAD_50_ of ASFV Arm07 within the right semimembranosus muscle. The animals were kept for 32 days post-challenge (dpc) or until they succumbed to the disease and humane endpoints were consequently established (see **Figure 1**). During the vaccination period, which can be understood as the period between the prime vaccination and the challenge (42 dpv), paired EDTA-blood and serum samples were taken once a week, on 0, 7, 11, 18, 25, 32 and 39 dpv for the detection of the ASF viral genome and its antibodies. During the challenge period, between 0 dpc/42 dpv until the end of the experiment (32 dpc/74 dpv), the sampling rate was maintained on 0, 4, 7, 11, 18, 25 and 32 dpc or the last day of each animal’s life.

### Clinical Evaluation

A clinical evaluation was performed on a daily basis in order to examine any clinical signs of development during the vaccination and challenge periods to evaluate the respective attenuation and effectivity of the vaccine prototype. The animals were observed on each day of the experiment utilizing a 24-h video camera and *in situ* wildlife-specialist veterinarian visits in order to record their daily clinical signs.

These clinical signs were expressed in terms of a quantitative CS following the specific guidelines for ASF clinical disease evaluation in wild boar previously described by Cadenas-Fernández et al. (18). This CS includes rectal temperature, behavior, body condition, skin alterations, ocular/nasal discharge, joint swelling, respiratory signs, digestive signs and neurological signs. The only clinical parameter that was not taken daily was that of rectal temperature in order to minimize the management of animals, and it was, therefore, measured only twice a week and in animals with any severe sign. Fever was defined as a rectal temperature of above 40.0°C.

Clinical evaluations were also substantial in order to ensure the welfare of the animals. The humane endpoint was pre-defined as animals with a CS > 18, and animals with severe clinical signs (level 4) of fever, behavior, body condition, respiratory and digestive signs for more than two consecutive days were also included, following the standards described (18). In addition, any animals undergoing unacceptable suffering without reaching the pre-defined humane endpoint were also euthanized based on veterinarian criteria.

### Sample Analysis

The serum samples were assayed using a commercial ELISA test to detect specific antibodies against ASFV-p72 (INGEZIM PPA Compac K3, Ingenasa, Madrid, Spain), following the procedure described by the manufacturer. The indirect immunoperoxidase test (IPT) was used for the analysis of serum. ASFV antibody titers were determined by means of end-point dilution using IPT, as performed by the European Union Reference Laboratory (19).

A High Pure PCR Template Preparation kit (Roche Diagnostics GmbH, Roche Applied Science, Mannheim, Germany) was used to extract DNA from EDTA-blood samples. The ASF viral genome obtained from blood (viremia) was amplified by employing the Universal Probe Library (UPL) real-time PCR protocol, using undiluted extracted DNA for each sample (20). The results were expressed in Cq values (equivalent to cycle threshold, CT), and were considered positive when Cq was < 40.0. Virus isolation was performed using PBM cells, as described in the Manual of Diagnostic Tests and Vaccines for Terrestrial Animals (21). The plates were examined for hemadsorption for six days and samples were blind-passaged three times on PBM. Real-time PCR was conducted after each isolation.

### Data Analysis

The statistical analysis was carried out using the SPSS 20 statistical program (IBM, Somar, NY, USA) and the R software, version 3.5.0 (22). A descriptive analysis of temperature, CS values, antibody response, the load of ASF viral genome (viremia; Cq values) in blood was performed in order to calculate average ranges per group and sampling period and at 95% confidence intervals. The variations in these parameters between groups and among different periods were studied using the Mann-Whitney U test and the Kruskal-Wallis test, respectively. Relationships among continuous parametric variables, temperature, CS and Cq values were statistically performed using Spearman’s rank correlations. Outcomes were considered statistically significant at p < 0.05.

## Results

### Vaccination Period

**Figure 2:**
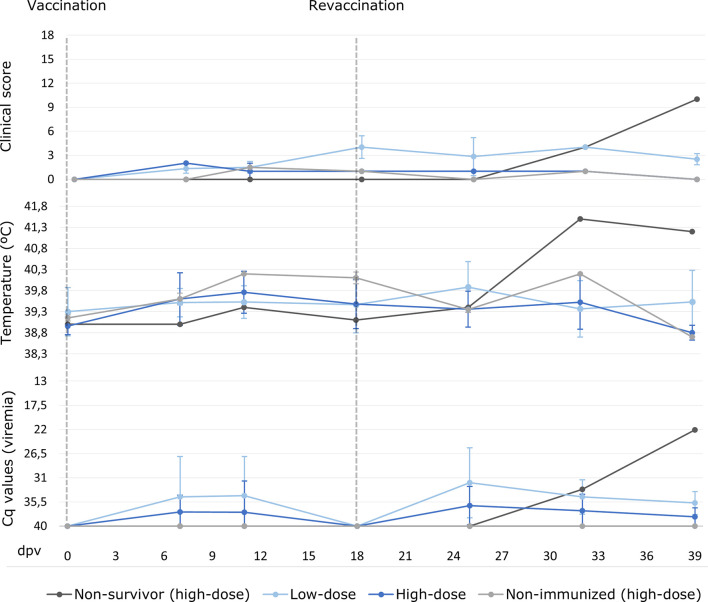
Averages of clinical score, rectal temperature and viremia expressed in cycles of quantification (Cq) values of real-time PCR carried out for wild boar orally vaccinated with 10^3^ TCID_50_ (low-dose group; light blue) and 10^4^ TCID_50_ of Lv17/WB/Rie1 ASFV (high-dose group; dark blue) on the sampling days post-vaccination (dpv). The results obtained for the non-surviving animal from the high-dose group during the vaccination period are presented separately (dark grey). Also, shown separately the two animals from the high-dose group that were non-immunized (light grey).

After the prime vaccination, two of the seven low-dose vaccinated animals (28.6%) developed a slight fever (40.45 ± 0.35°C; **Figure 2**) and slight lethargy from 11 and 18 dpv, respectively, and this was observed on only one sampling day. After the revaccination, these two, plus three more animals from this group, subsequently had a slight fever (40.50 ± 0.29°C; **Figure 2**) at 12 days post revaccination (25 – 39 dpv). Two of these animals had a slight fever on two consecutive sampling days, during which time slight lethargy was also observed in addition to fever. All the vaccinated animals from the low-dose group recovered and survived in the vaccination period before the challenge, and the highest clinical score (CS) recorded was 5.5 at 25 dpv (7 days post-revaccination). The mean CS during this period was, overall, 0.7 ± 1.4 (**Figure 2**).

With regard to the high-dose group, after prime vaccination, only three out of six animals (50%) developed a slight fever (40.15 ± 0.10°C; **Figure 2**) from 11 dpv, and this was observed on two consecutive sampling days. After the revaccination, one of these animals and another had a slight fever (40.10 ± 0.14°C; **Figure 2**) at 10 days post revaccination (25 – 32 dpv), and one of them also had slight lethargy. Moreover, one wild boar from this group had a high fever (41.5°C) at 14 days post revaccination (32 dpv). This animal was the only vaccinated animal that succumbed during the vaccination period, since it developed a clinical course that began after a highly aggressive fight inside the pen. This fight resulted in multiple external injuries and the dislocation of the animal’s right hip. After this fight, the animal was treated in the pen in order to aid its recovery, but began to show signs of fever followed by lethargy and finally anorexia, until its euthanasia at 21 days post revaccination (39 dpv), with an ASF CS of 10. The analysis of the samples obtained revealed the presence of ASF viral DNA in blood along with septicemia owing to *Streptococcus suis* and the detection of the DNA bacteria in the brain. The other animals in the high-dose group recovered and survived in the vaccination period, and the highest recorded CS, apart from that of the animal which did not survive, was 2 at 7 dpv. The mean CS during this period was, overall, 0.5 ± 1.2 (**Figure 2**).

No statistically significant differences were observed between the low-dose and high-dose groups during the vaccination period in terms of temperature variations and CS (Mann-Whitney U test, p = 0.25, p = 0.12, respectively).

Two transient peaks of ASF viral genome detection in blood were generally observed in all the vaccinated animals. The first peak was observed after the prime vaccination in five of the 13 animals (38.5%), while the second was observed after revaccination in 11 animals, which is in 84.6% of them (see **Figure 2**). Furthermore, a direct correlation was observed between the viremia peaks and the increase in rectal temperature (Spearman’s rank, p< 0.05, r = - 0.37).

After the prime vaccination, the ASF viral genome was detected transiently in the blood of three wild boar from the low-dose group, starting from 7 dpv with a mean Cq value of 27.1 ± 4.0; only one of these animals had a fever before the revaccination. After the prime vaccination, two wild boar in the high-dose group started to show a positive viremia from 7 dpv, with mean Cq values of 32.1 ± 4.5, and one of these animals had a fever before the revaccination. After the revaccination, the ASF viral genome was detected transiently in the blood of the aforementioned animals, in that of the other animals from the low-dose group and in two more wild boar from the high-dose group, starting from 7 to 21 days post revaccination (25 – 39 dpv; mean Cq values of 32.9 ± 3.7 and 34.4 ± 4.7, respectively). There were no statistically significant differences as regards viremia between the low-dose and high-dose groups during the entire vaccination period (Mann-Whitney U test, p = 0.16).

**Figure 3:**
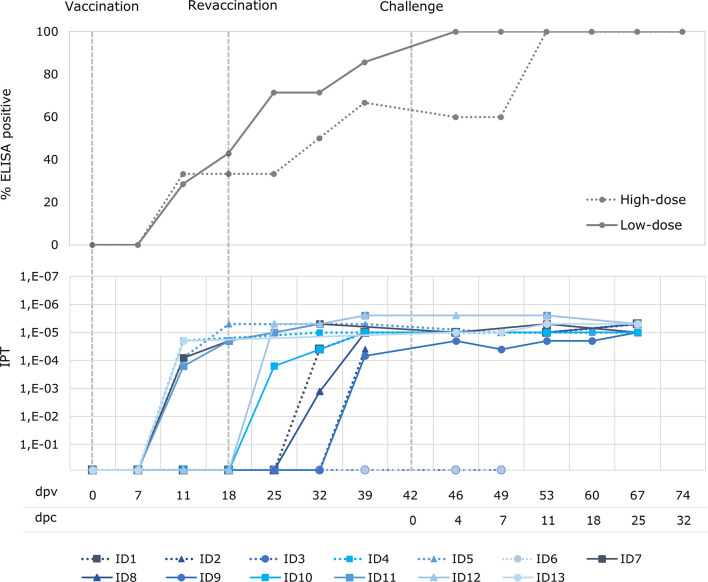
Percentage of wild boar with positive antibody response (ELISA) and titers of antibodies (indirect immunoperoxidase test; IPT) after oral vaccination with 10^3^ TCID_50_ (low-dose group; unbroken lines) and 10^4^ TCID_50_ of Lv17/WB/Rie1 ASFV (high-dose group; dotted lines) after vaccination (dpv) and challenge (dpc).

Before the challenge, all the animals from the low-dose group had a positive ASFV antibody response, obtained using both the ELISA and the IPT, with the exception of one that tested antibody positive only for IPT (**Figure 3**). The same three animals from the low-dose group that had viremia before the revaccination were positive to ASFV antibodies before the revaccination, from 11 dpv, and the four remaining animals began to show a positive antibody response from 7 to 21 days post revaccination (25 – 39 dpv). With regard to the high-dose group, four out of six animals (66.7%) had a positive ASFV antibody response according to both the ELISA and IPT tests. The same occurred with the two animals from the high-dose group, which had viremia before the revaccination and were positive to ASFV antibodies before the revaccination from 11 dpv, and the two remaining antibody-positive animals, starting from 14 to 21 days post revaccination (32 – 39 dpv). The antibody-positive animals maintained a high titer of antibodies throughout the experiment (**Figure 3**).

There was a clear direct correlation between the start of a positive ASFV antibody response and the first detection of ASF viral genome (Spearman’s rank, r = 0.99; p< 0.05),. Indeed, the animals from the high-dose group in which viremia was not detected coincided with the two animals in this group in which no antibody response was detected.

### Challenge Period

All the animals from the low-dose group were fully protected and survived the challenge with Arm07 10 HAD_50_ at 32 dpc (100% protective efficacy). However, the two high-dose vaccinated animals that did not have a positive antibody response during the vaccination period did not survive after the challenge (hereafter, unprotected; 60% protective efficacy in this group), and had the same ASF-compatible signs as those observed in the control group. The protection outcome for both groups of wild boar orally vaccinated and revaccinated with Lv17/WB/Rie1 ASFV was, overall, 83.3%.

**Figure 4:**
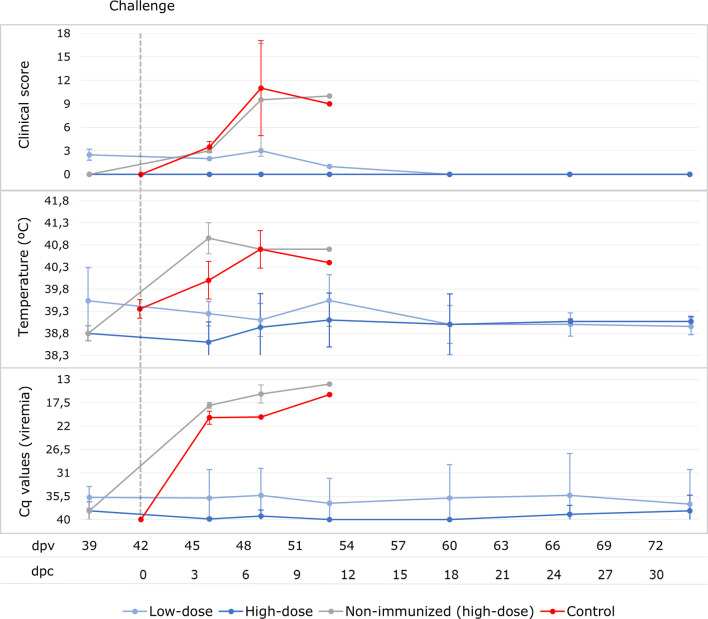
Averages of clinical score, rectal temperature and viremia expressed in Cq values of real-time PCR carried out for wild boar orally vaccinated with 10^3^ TCID_50_ (low-dose group; light blue) and 10^4^ TCID_50_ of Lv17/WB/Rie1 ASFV (high-dose group; dark blue) and two naïve wild boar (control; red) after the IM challenge with 10 HAD_50_ of Arm07 at 42 days post-vaccination (dpv) at the sampling days post-challenge (dpc). The results obtained for the two non-surviving animals from the high-dose group after the challenge are presented separately (light grey).

No clinical signs were observed in the low-dose group after the IM challenge, except for a very slight rise in rectal temperature in one animal (40.2°C) at 11 dpc (see **Figure 4**). With regard to ASF viral genome detection in blood, five animals from this group had a transient viremia after the challenge, starting from 4 dpc (46 dpv) with a mean Cq value of 36.4 ± 2.0 (see **Figure 4**), while only one animal maintained constant viremia from 4 dpc until the end of the experiment, with a mean Cq value of 25.5 ± 0.8. In this respect, none of the survivors from the high-dose group developed any clinical signs after the challenge, and there was not even a slight rise in rectal temperature. Only one wild boar in this group had a transient viremia after challenge, with a mean Cq value of 37.4 ± 2.0 (see **Figure 4**).

The two unprotected wild boar from the high-dose group developed a fever of 40.7 - 41.2°C (see **Figure 3**) at 4 dpc, followed by lethargy, anorexia, erythema and respiratory difficulties. These animals never had a positive antibody response, rapidly reached a CS of 16 (7 dpc) and were consequently euthanized at 7 and 10 dpc (49 and 52 dpv), respectively.

This clinical course was similar to that observed in the control animals, which were also euthanized at 5 and 7 dpc, respectively, after reaching a CS of 18. All challenged control and unprotected vaccinated animals had a high viremia (Cq= 16.9 ± 1.7 and 19.3 ± 2.3, respectively), which started at 4 dpc and continued until death (see **Figure 3**). The viremia, temperature and CS were, overall, significantly higher in the challenged control and unprotected animals than in the vaccinated survivors (Mann-Whitney U test, p < 0.05).

### Post-Mortem Studies

The challenged controls and unprotected animals from the overdose group showed pathological lesions consistent with ASF, confirmed by qPCR, virus isolation and hemadsorption. The main necropsy findings were moderate to severe accumulation of yellowish to reddish fluid in the abdominal cavity (ascites), thorax (hydrothorax), and pericardial sac (hydropericardium). Pulmonary oedema, congestion, and multifocal haemorrhages on the surface of the lung have been observed. There was congestion and enlargement of the spleen (splenomegaly), liver (hepatomegaly) and lymph nodes (lymphadenomegaly). In the latter, hemorrhages of varying degrees of severity were observed, as well as in the kidney and intestine mucosa (**Supplementary Material**). All tissues (100%) from these animals were positive to ASF viral genome (20 different tissues). The mean Cq values from the tissues of the control animals was 22.8 ± 2.3, similar to the mean of the tissues of the unprotected animals of the overdose group (Cq = 20.3 ± 2.5).

On the other hand, all vaccinated animals within the low-dose and high-dose groups which survived the challenge did not show any lesion compatible with ASF at 32 dpc (**Supplementary Material**). On average, only 7 ± 3 tissues (35%) of wild boar from these groups were positive to ASF viral genome. ASFV could be isolated from only one retropharyngeal lymph node of all 20 tissues analyzed from these animals. This virus isolate was non-hemadsorbing. The mean Cq value from tissues in the survived animals from low-dose group was 37.1 ± 0.5, very similar to the mean Cq value from tissues in the survived animals from high-dose group, 36.3 ± 2.0. ASF viral genome levels of these groups were significantly lower (Cq = 38.8 ± 1.9) than in challenged controls and unprotected animals from high-dose group (Mann-Whitney U test, p < 0.05).

## Discussion

The viral replication properties of attenuated vaccines make them a great tool for the effective control of various diseases (23). Although they have commonly been described as insufficiently safe against ASFV owing to chronic clinical forms, side-effects and non-sterile immunity (24), our clinical trial provided promising results in terms of innocuousness and safety in orally vaccinated wild boar. However, there was a relevant difference in terms of safety depending on the dosage used for vaccination. Two of the seven low-dose animals and two of the five high-dose animals that survived did not show any clinical signs after prime and re-vaccination. The remaining animals had only a slight transient fever, along with slight lethargy, similar to observations attained previously after an oral vaccination (12). Nevertheless, one of the high-dose vaccinated wild boar got worse after a hierarchical fight inside the pen that coincided with the revaccination and subsequently died.

With regard to effectivity, all the animals with a positive antibody response before the challenge (100% of the low-dose and 60% of the high-dose group) were fully protected. Overall, the results determined that the vaccine candidate protected 83.3% of wild boar against the challenge with the virulent ASFV genotype II Arm07 isolate. This result not only shows the animals’ survival when confronted with the challenge but also the absence of clinical signs. The reason for testing this vaccine in revaccination and with an high-dose is that of mimicking the administration of oral vaccine baits and their consumption by wild boar in the field, as has occurred previously in successful experiences concerning the oral immunization of wild boar against classical swine fever in Germany and against tuberculosis in Spain (25, 26).

The orally vaccinated animal that succumbed after a highly aggressive fight inside the pen creates major unknowns and concerns that must, therefore, be addressed in detail in order to decipher the cause of the death and the implication of the Lv17/WB/Rie1 isolate as well as to discover what triggers this lethal outcome in sporadically vaccinated animals so as to assess whether it can be prevented and controlled. It is also necessary to consider that these animals were subjected to variable levels of stress during the experiment, since wild animals are not used to the proximity of humans and handling. Moreover, these experiments were carried out with groups of wild boar sharing the same pen, which gave rise to fights. Although all these contemplations were considered and controlled throughout the experiment, it was inevitable that the animals would develop a certain level of stress. This level of stress, which led to immunosuppression (26), along with exposure to the vaccine isolate or even other opportunistic pathogens, may have triggered the lethal outcome that we observed in one of the orally vaccinated animals.

We think that this important safety unknown and concern is related to high doses and certain circumstances of stress, as is this case belonging to the study group that received the highest dose and was injured after a fight, but does not apply to low-dosed or healthy animals. Although further studies are needed in this regard, since no statistically significant differences in innocuousness were observed between the rest of the vaccinated animals with high doses (5/6 animals) compared to the low-dose vaccinated (7/7 animals) in terms of rectal temperature, CS and viral genome load in blood. These animals developed only a transient fever along with slight lethargy, which is in line with the results previously obtained with this vaccine candidate (12). The transient fever was directly correlated with the two peaks of viremia, one after vaccination and the other after revaccination, as has also been described for other attenuated vaccine candidates as regards both naturally or artificially genetic deletion (27, 28). Moreover, these slight temperature peaks may even be due to stress reactions or other opportunistic or latent infectious diseases.

For all this, we suggest carrying out further dose adjustment studies with doses that are even lower than the current low-dose, since the effectiveness does not appear to be affected by the dosage. Additionally, it would also be interesting to test even higher doses to check the relationship between vaccine safety and dose. The evaluation of high doses for this vaccine prototype is essential for two reasons; owing to it is a live attenuated vaccine and the other is that wild boar is the target, which has to be vaccinated orally from baits. Under these conditions, the control of the dose per individual in the field is much more complex than at the level of domestic animals, so we have to take into account the risk of animals taking even much higher doses. In addition, it is likely that we cannot define a very low dose due to the same fact that the vaccination is done from baits thrown in the field, which can be there for hours or even days and this can affect the viability of the vaccine and its effectiveness. To clarify this issue, studies are needed to evaluate the stability of the virus in the bait under different environmental conditions and at different times.

Viremia and, therefore, viral replication would appear to be essential for the development of a protective immune response against ASFV infection (28, 29), since a direct correlation was observed between viremia and the appearance of antibodies responses. Although there is a lack of knowledge and there are contradictions regarding the specific immune mechanisms that are involved in the protective response against ASFV infection (24), the appearance of antibodies has, in the current study, been indicative of effective protection against a challenge. Since the only two vaccinated animals that were not protected against the challenge were also the only vaccinated animals that did not attain a positive antibody response, this is in line with previous studies (12). Interestingly, these two vaccinated and unprotected animals belonged to group 10^4^ TCID_50_. This result partially contrasts with the previous study (12), in which the oral vaccine at this dose, without revaccination, conferred 92% protection against the challenge with the Arm07 isolate virus, but by means of contact with infected animals (i.e., the shedder-pig challenge-exposure infection model). In this previous study, three animals did not initially react to the oral vaccine (no fever, viremia or antibody response), but were, upon contact (likely oral) with the virulent isolate, protected, and provided positive results as regards antibody response a week after the challenge. In the current study, however, the two animals that did not initially react to the oral vaccination were exposed to an intramuscular challenge. This difference in results could be explained by the different ways in which the animals were exposed to the virulent virus; an oral *versus* an intramuscular vaccination. These results also highlight the potential importance of mucosal-associated immunity against this infection. More studies highlighting contact/natural challenge are required, since immunization is performed orally. This would allow us to understand and evaluate the effect of the mucosal barriers, previously activated by oral immunization, against ASFV infection.

The importance of viral replication in the development of a protective immune response against ASFV is also supported by the recent and historical ASF vaccine trials, since these studies have shown that both inactivated and subunit vaccines have a lack of protection (19, 30–32), while attenuated vaccines, either naturally (13, 16, 18, 33, 34) or by genetic deletion (34–37), have shown very promising results regarding protection, which are close to 100%. This relevance of viral replication supports the idea that the cellular immune response plays a key role in protection against ASFV since this response needs viral replication (38, 39), which is also in line with the controversy about the role of antibodies in the protective immune response against this pathogen (24). Although antibodies against ASFV have partially demonstrated neutralizing activity in assay cultures (39), these results do not correspond to those observed *in vivo*. Passive antibody transfer studies have shown partial protection against ASFV (40–42), but many other studies have shown that the presence of antibodies is not sufficient to confer a protective immune response (43, 44), so antibodies against ASFV are not considered to be fully neutralizing.

It is important to note that not all the vaccinated and protected animals developed viremia and antibodies before revaccination, and it is, therefore, not known whether they would have developed a protective immune response only after the initial vaccination. Possibly, oral immunization may not work in all cases compared to intramuscular administration (e.g. in domestic swine) (15). Hence the importance of evaluating the efficacy of repeated vaccinations by oral route, to improve that immunization rate. Further studies on this are, therefore, required, because this is a key aspect as regards making decisions concerning the design of oral vaccination campaigns in the field.

As mentioned previously, the two vaccinated animals that were unprotected during the challenge were from the high-dose group. This means that, in this case, the dose does not appear to be related to the effectiveness of the vaccine candidate tested. Keeping this in mind, along with the fact that the vaccinated animal that did not survive before the challenge was also from this high-dose group, it would be very interesting to carry out further studies with doses even lower than 10^3^ TCID_50_. This would allow us to adjust the dose for it to be safer without losing effectiveness.

Although there are still concerns about the safety of this vaccine prototype, since it is a naturally attenuated live vaccine, there are many compelling reasons to continue studying it and to seek ways in which to make it safer, the reason being that it is the only vaccine prototype that has had highly effective results in wild boar by oral administration (12) to have been published to date. Having a tool like this candidate vaccine for the control of ASF in wild boar would greatly help control the spread of the disease in these populations. Cannibalism could be one of the mechanisms of ASFV transmission among wild boar populations (45, 46). If wild boar were protected with this vaccine, not only would the mortality rate decrease considerably, but also the levels of the virus in the tissues. In addition, previous studies have determined that orally vaccinated animals do not excrete or excrete very low levels of the vaccine isolate (46).

This strategy has been observed naturally in wild boar populations in which the virus has remained for a long time and has evolved into less virulent isolates. A greater number of animals with positive antibodies but negative to viremia, and consequently without active infection, have been found (47).

Overall, after the vaccination and the revaccination at 18 dpv with the vaccine prototype Lv17/WB/Rie1, the wild boar vaccinated with a low-dose were no different from those that underwent a low vaccination in the previous study in terms of safety. The exception is the high-dose animal that succumbed after revaccination under the conditions mentioned above, with the vaccine isolate being innocuous for low-dose and healthy animals, which developed only a slight transient fever and viremia (12). The fatality after revaccination and before challenge raises concerns and the need for further studies. However, there is the scope to test lower and consequently safer doses, because effectiveness was not compromised by the reduction in dose. Considering all these results and the epidemiological situation of ASF in wild boar, this vaccine prototype is still a promising tool for the control of the disease in these wild populations, although there is a great need for further studies.

## Data Availability Statement

The original contributions presented in the study are included in the article/**Supplementary Material**. Further inquiries can be directed to the corresponding authors.

## Ethics Statement

The animal study was reviewed and approved by Ethics Committees of the Madrid Complutense University and the Community of Madrid (reference PROEX 159/19).

## Author Contributions

JB, EC-F, and JS-V participated in the experimental design. BR and CG prepared the viral inoculations. JB, EC-F, AK, SB-A, BR, RS, NP, and CG conducted the field and laboratory work. JB, EC-F and JS-V performed the data analysis and visualization. JB and EC-F drafted the manuscript. JB, EC-F, AK, SB-A, BR, RS, NP, CG, and JS-V revised and approved the manuscript. All authors contributed to the article and approved the submitted version.

## Funding

This research was funded by the H2020 VACDIVA 862874 project. EC-F is a recipient of a Spanish Government-funded PhD fellowship for the Training of Future Scholars (FPU) awarded by the Spanish Ministry of Education, Culture and Sports.

## Conflict of Interest

The authors declare that the research was conducted in the absence of any commercial or financial relationships that could be construed as a potential conflict of interest.

## Publisher’s Note

All claims expressed in this article are solely those of the authors and do not necessarily represent those of their affiliated organizations, or those of the publisher, the editors and the reviewers. Any product that may be evaluated in this article, or claim that may be made by its manufacturer, is not guaranteed or endorsed by the publisher.
