# Supplementary material for: Safety of African Swine Fever Vaccine Candidate Lv17/WB/Rie1 in Wild Boar: Overdose and Repeated Doses
Source: Front Immunol. 2021 Nov 30;12:761753. doi: 10.3389/fimmu.2021.761753 (PMC8669561; doi:10.3389/fimmu.2021.761753)
Supplement: Supplementary file 1 [file DataSheet_1.docx]

Supplementary Material


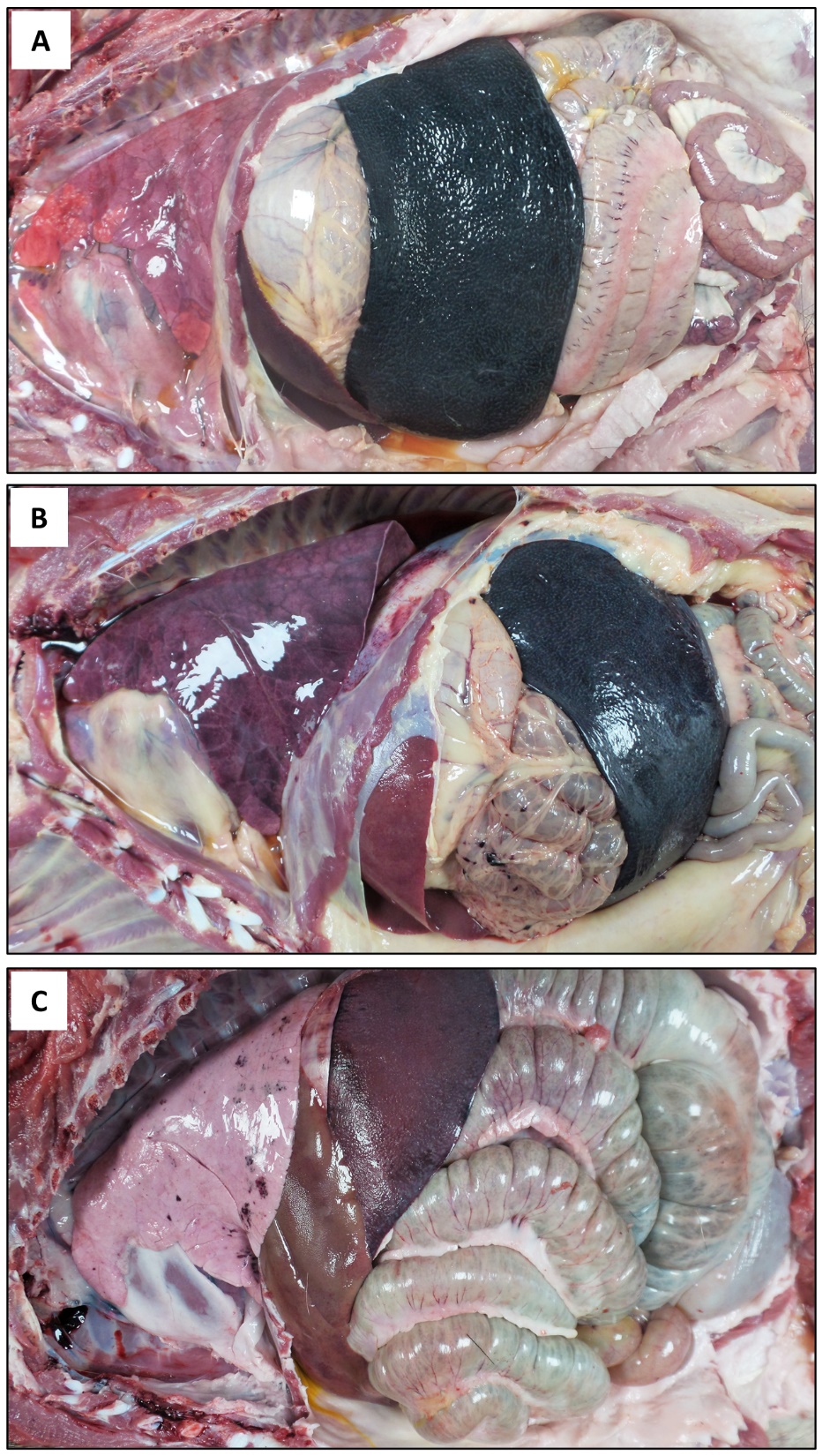


**Supplementary Figure 1.** Necropsy findings. Views of thoracic and abdominal cavities from (A) control wild boar (naïve animal IM challenged with Arm07), (B) orally vaccinated wild boar with Lv17/WB/Rie1 that did not survive the challenge, and (C) orally vaccinated wild boar with Lv17/WB/Rie1 that survived the challenge. Splenomegaly, congestive spleen, hydrothorax, and pulmonary edema and congestion are evident in (A) and (B).
